# Omic Profiling of Extracellular Vesicles from Two Cord-Related Sources Reveals Divergent Effects on Melanogenesis

**DOI:** 10.3390/cimb48040391

**Published:** 2026-04-10

**Authors:** Chia-Ni Hsiung, Wen-Yu Lien, Martin Sieber, Wen-Hsien Lin

**Affiliations:** 1 GGA Corp., Taipei 114065, Taiwan; 2 BIONET Therapeutics Corp., Taipei 114065, Taiwan; 3 BIONET Corp., Taipei 114065, Taiwan

**Keywords:** extracellular vesicles, umbilical cord mesenchymal stem cells, cord blood plasma, miRNA profiling, proteomics, pigmentation regulation, melanogenesis, AI-driven analysis

## Abstract

Extracellular vesicles (EVs) mediate intercellular communication by delivering proteins and RNAs, with their molecular cargo often reflecting the biological context of their source. Perinatal tissues are promising sources of EV-related biomaterials with potential dermatologic applications. In this study, we compared EV-related molecular cargo from two umbilical cord-associated sources, umbilical cord mesenchymal stem cell (UCMSC)-derived EVs and cord blood plasma (CBP), to investigate whether these materials exhibit distinct functional effects on melanogenesis. UCMSC-derived EVs were isolated from conditioned culture medium and characterized using nanoparticle tracking analysis (NTA), cryo-electron microscopy (cryo-EM), and canonical EV marker detection, while cord blood samples were processed to obtain plasma following centrifugation and filtration, containing EVs together with soluble plasma components. Functional assays in the murine melanocyte cell line B16F10 demonstrated that UCMSC-derived EVs suppressed melanin production, whereas CBP treatment enhanced melanogenesis. Integrative omics analyses combining microRNAs (miRNAs) microarray profiling and proteomic characterization revealed distinct molecular signatures between UCMSC-derived EVs and CBP samples. Functional validation using miRNA mimic assays showed that selected miRNAs, including miR-6862-5p, miR-3622b-5p, miR-7847-3p, miR-6774-5p, and miR-4685-5p, reduced melanin production, whereas others, including miR-203a-3p, miR-126-3p, miR-139-5p, and miR-15b-5p, increased melanin levels. Pathway analysis using Ingenuity Pathway Analysis (IPA) (QIAGEN Inc.) associated these miRNA subsets with signaling pathways involved in melanogenesis. Together, these findings indicate that UCMSC-derived EVs and CBP exhibit opposite functional effects on melanogenesis and possess distinct miRNA and protein cargo profiles, providing potential molecular targets for modulating pigmentation and supporting the development of EV-related therapeutic strategies for pigmentation disorders.

## 1. Introduction

EVs are a heterogeneous group of cell-derived, membrane-enclosed particles that play a crucial role in intercellular communication by transferring a diverse cargo of proteins, lipids, and nucleic acids, including miRNAs [1,2]. Their ability to modulate recipient cell function has positioned them as key players in numerous physiological and pathological processes, as well as promising candidates for therapeutic applications [3]. Among the various sources of EVs, mesenchymal stem cells (MSCs) have garnered significant attention due to their regenerative and immunomodulatory properties, which are largely mediated by their secreted EVs [4].

Human UCMSCs and CBP are perinatal materials within the same umbilical-cord unit. Operationally, they represent different sample types—adherent stromal cell cultures versus the cell-free plasma fraction. CBP naturally contains EVs together with abundant soluble proteins and other circulating biomolecules. We therefore adopt neutral terminology and investigate whether cargo and functional effects differ between these two cord-related sources. EVs derived from these sources are being explored for various applications, particularly in dermatology and regenerative medicine. For instance, MSC-derived EVs have been shown to promote wound healing, reduce scarring, and exhibit anti-aging effects [5,6]. Specifically, their role in skin pigmentation, or melanogenesis, is an area of growing interest. Some studies suggest that MSC-derived EVs can have a skin-lightening effect by inhibiting melanin production [7,8], while others report different or context-dependent outcomes.

Melanogenesis is the complex process of melanin synthesis within specialized organelles called melanosomes in melanocytes. This process is regulated by a network of signaling pathways and transcription factors, with the microphthalmia-associated transcription factor (MITF) acting as a master regulator that controls the expression of key melanogenic enzymes like tyrosinase (TYR), tyrosinase-related protein 1 (TYRP1), and tyrosinase-related protein 2 (TYRP2) [9]. Dysregulation of this process can lead to pigmentation disorders, ranging from hyperpigmentation (e.g., melasma) to hypopigmentation (e.g., vitiligo). Consequently, modulating melanogenesis is a primary goal in both cosmetic dermatology and the treatment of these disorders [10].

miRNAs, small non-coding RNAs of ~22 nucleotides, are critical post-transcriptional regulators that typically bind to the 3′-untranslated region (3′-UTR) of target messenger RNAs (mRNAs), leading to their degradation or translational repression [11]. Exosomal miRNAs are particularly important as they can be transferred between cells, acting as paracrine signaling molecules to modulate gene expression in recipient cells [12]. The specific miRNA cargo of an EV is a reflection of its cell of origin and can dictate its functional effect. In the context of melanogenesis, several miRNAs have been identified as either positive or negative regulators by targeting key components of the pigmentation pathway [13,14].

Given the distinct cellular origins of UCMSCs and CBP, we hypothesized that their respective EV populations would possess different molecular cargos and, consequently, exert divergent functional effects on melanogenesis. In this study, we performed a comprehensive characterization and functional comparison of UCMSC-derived EVs and CBP. We discovered that they indeed have opposing effects: UCMSC-derived EVs inhibit melanogenesis, while CBP promotes it. To unravel the molecular mechanisms underlying this functional dichotomy, we conducted an integrative omics analysis, combining proteomics and miRNA profiling. We utilized a suite of AI-driven bioinformatic tools to predict miRNA–target interactions and pathway analyses to identify the key molecular players responsible for these opposing biological activities. Our findings not only shed light on the distinct regulatory roles of EVs from different cord-related sources but also identify specific miRNA and protein signatures that could be harnessed for therapeutic interventions in pigmentation disorders.

We first conducted unbiased, global analyses of EV miRNA and proteome profiles from UCMSC-derived EVs and CBP, which revealed distinct clustering between the two sources in both Principal component analysis (PCA) and heatmap analyses. Among enriched biological themes, pigmentation/melanogenesis emerged with both statistical support and coherent directionality across molecules. We therefore prioritized melanogenesis for focused validation, given its clear translational relevance to hyper- and hypopigmentation.

This study, therefore, aimed to determine whether UCMSC-derived EVs and CBP exert distinct effects on melanogenesis and to delineate the associated miRNA–target networks through an AI-assisted, multi-omics integration approach. Our findings provide novel insights into the source-dependent functional diversity of perinatal EVs and their potential applications in modulating pigmentation for therapeutic and cosmetic purposes.

## 2. Materials and Methods

### 2.1. Cell Culture

Murine melanoma B16F10 cells were purchased from the Bioresource Collection and Research Center (BCRC), BCRC 60031, Hsinchu, Taiwan. B16F10 mouse melanoma cells were cultured in Dulbecco’s Modified Eagle’s Medium (DMEM) supplemented with 10% fetal bovine serum (FBS) and 1% penicillin/streptomycin. Cultures were maintained in a humidified air incubator containing 5% CO_2_ at 37 °C.

UCMSCs were isolated from the intervascular Wharton’s jelly of human umbilical cords after obtaining informed donor consent. Fresh umbilical cord tissues were processed under sterile conditions, and Wharton’s jelly was dissected and cut into small pieces for explant culture. The tissue fragments were plated as whole explants to allow spontaneous migration of MSCs. The primary culture was maintained for 7–14 days until sufficient cell outgrowth was observed.

The outgrown cells exhibited a typical spindle-shaped, fibroblast-like morphology. UCMSCs were cultured in Minimum Essential Medium Alpha (MEMα; Gibco, Grand Island, NY, USA, 41061029) supplemented with 5% UltraGRO (AventaCell Biomedical, New Taipei City, Taiwan) and maintained in a humidified incubator at 37 °C with 5% CO_2_. The culture medium was replaced every 2–3 days, and cells were passaged upon reaching approximately 80–90% confluency.

Cells at passages 3–5 were used for all subsequent experiments. For EV collection, UCMSCs were cultured in serum-free MEMα for 48 h. The conditioned medium was then collected and processed for EV isolation.

### 2.2. EV Isolation

For the isolation of UCMSC-derived EVs, the conditioned medium was collected and concentrated using Macrosep™ centrifugal filters (MWCO 3K; PALL, Marlborough, MA, USA, MAP100C38) by centrifugation at 2330× *g* for 50 min at room temperature. Concentrate was then sterile filtered using a 0.2 μm disc filter (PALL, Marlborough, MA, USA, 4602).

For CBP preparation, donated human umbilical cord blood was centrifuged at 400× *g* for 20 min to separate plasma. After centrifugation, the upper plasma layer was collected and sterile filtered using a 0.2 μm disc filter. This preparation is referred to as CBP and was used directly in subsequent experiments. CBP contains EVs together with soluble plasma components.

### 2.3. UCMSC and EV Characterization

The expression of MSC surface markers in UCMSCs was evaluated by flow cytometry. Cells were washed with Dulbecco’s phosphate-buffered saline (DPBS; Gibco, Grand Island, NY, USA) and incubated for 30 min at room temperature in the dark with fluorescein isothiocyanate (FITC)- or phycoerythrin (PE)-conjugated antibodies (BD Biosciences, San Jose, CA, USA) against CD13, CD29, CD44, CD73, CD90, and CD105, as well as hematopoietic and endothelial markers CD14, CD19, CD31, CD34, CD45, and HLA-DR. Isotype-matched antibodies were used as controls. After incubation, cells were washed and subjected to flow cytometric analysis. A total of 1 × 10^4^ events were acquired for each sample and analyzed using FACS Lyric software, version 1.4.1 (Becton Dickinson, Franklin Lakes, NJ, USA).

To evaluate multipotent differentiation capacity, UCMSCs were cultured in adipogenic, osteogenic, and chondrogenic differentiation media (StemPro differentiation kit, Gibco, Grand Island, NY, USA) according to the manufacturer’s instructions. Cells were maintained under differentiation conditions for 14–21 days, with medium changes performed as recommended. Following induction, adipogenic, osteogenic, and chondrogenic differentiation were assessed by Oil Red O, alkaline phosphatase (ALP), and toluidine blue staining, respectively.

For cryo-EM, Quantifoil R1.2/1.3 300-mesh grids (Sigma-Aldrich, St. Louis, MO, USA) coated with a 2 nm continuous carbon film were glow-discharged using a GloQube Plus system (Quorum, San Jose, CA, USA) for 80 s at 30 mA. A 4 µL drop of exosome sample was applied to the grids and blotted for 2 s using a Vitrobot Mark IV (Thermo Fisher Scientific, Waltham, MA, USA) at 4 °C, 100% humidity, and a blot force of −4. Grids were then rapidly plunged into liquid ethane cooled by liquid nitrogen. Movies were acquired on a Glacios Cryo-EM (Thermo Fisher Scientific, Waltham, MA, USA) operated at 200 kV with a Falcon 4 detector using EPU software, version 3.11. Images were recorded at 92,000× magnification in counting mode (1.6 Å/pixel), and Feret’s diameters were analyzed using ImageJ, version 1.53c (NIH, Bethesda, MD, USA).

The size distribution and concentration of EVs were determined using a ZetaView PMX 230 NTA system (Particle Metrix, Inning am Ammersee, Germany) and analyzed with ZetaView software, version 8.06.01 SP1.

The presence of EV surface markers was confirmed by flow cytometry. EVs were captured using the Exosome-Human CD81 Flow Detection Reagent (Invitrogen™, Waltham, MA, USA, 10622D) and subsequently stained with PE-conjugated antibodies against CD9 (BD Pharmingen, San Diego, CA, USA, 555372), CD63 (BD Pharmingen, San Diego, CA, USA, 556020), and CD81 (BD Pharmingen, San Diego, CA, USA, 555676). PE-conjugated IgG1κ (BD Pharmingen, San Diego, CA, USA, 555749) served as the isotype control. Fluorescence signals were analyzed using a FACSLyric™ flow cytometer (BD Biosciences, San Jose, CA, USA).

### 2.4. miRNA Profiling

Total miRNA was extracted using the miRNeasy Mini Kit (QIAGEN, Hilden, Germany, 217004), and RNA concentration was measured spectrophotometrically. Samples were stored at −20 °C until use. For microarray analysis, miRNAs were labeled with the FlashTag™ Biotin HSR RNA Labeling Kit (Thermo Fisher Scientific, Waltham, MA, USA, 901911) according to the manufacturer’s instructions, including poly(A) tailing and biotin ligation steps. RNA Spike Control Oligos were used to monitor labeling and hybridization efficiency. Labeled RNA was hybridized to the GeneChip™ miRNA 4.0 Array (Thermo Fisher Scientific, Waltham, MA, USA) at 48 °C for 16–18 h, followed by washing and staining on the GeneChip^®^ Fluidics Station 450 and scanning with the GeneChip^®^ Scanner 3000. Raw CEL files were processed in R (version 4.4.1), and only miRNAs with signal intensities above the median of each array were included in subsequent pathway analysis. Biological replicates were UCMSC-derived EVs (*n* = 3) and CBP (*n* = 3); donor numbers were UCMSC donors = 10 and CBP donors = 3.

### 2.5. Protein Profiling

Total protein was extracted using RIPA buffer containing protease and phosphatase inhibitors. Protein concentration was determined with the BCA Protein Assay Kit (Thermo Fisher Scientific, Waltham, MA, USA), and samples were stored at −80 °C until analysis. Proteins were reduced with DTT, alkylated with iodoacetamide, and digested with trypsin overnight at 37 °C. Peptides were desalted using C18 cartridges and analyzed by LC–MS/MS on an Orbitrap Fusion Lumos Tribrid mass spectrometer (Thermo Fisher Scientific, Waltham, MA, USA) coupled to an UltiMate 3000 nanoLC system (Thermo Fisher Scientific, Waltham, MA, USA). MS data were searched against the UniProt Human database using Mascot 2.3 and quantified in Proteome Discoverer 2.5 with a false discovery rate (FDR) < 1%.

### 2.6. AI-Driven Bioinformatic Analysis and miRNA Target Prediction

To identify miRNAs potentially regulating melanogenesis, we employed a multi-tiered bioinformatic pipeline. First, differentially expressed miRNAs from the GeneChip microarray data were filtered. Then, several AI-driven algorithms were used to predict the mRNA targets of these miRNAs. The rationale was to leverage the strengths of different prediction models to generate a high-confidence list of miRNA–target interactions relevant to pigmentation.

The prediction process involved tools such as miTAR, a hybrid deep learning model combining convolutional and recurrent neural networks (CNNs and RNNs) to learn spatial and sequential features from raw sequences [15]; miRAW, a deep learning tool that analyzes the entire miRNA transcript to identify both canonical and non-canonical binding sites [16]. Unlike seed-region-based prediction methods, miRAW analyzes the entire miRNA transcript using deep learning, enabling the identification of both canonical and non-canonical binding sites. This capability expanded the candidate target repertoire to include regulatory interactions that would be missed by conventional seed-matching algorithms, such as the reported non-canonical targeting of KIF5b by miR-203 in melanosome transport regulation [17]. DeepMirTar, which uses stacked denoising autoencoders and a comprehensive feature set for prediction [18]. The predicted target genes were cross-referenced with known melanogenesis-related genes from databases like KEGG and Gene Ontology. This integrated approach allowed for the selection of high-priority candidate miRNAs for further validation (summarized in Table 1). Target prediction was performed using miTAR (v1.0; https://github.com/tjgu/miTAR) (accessed on 25 March 2025), miRAW (https://bitbucket.org/bipous/workspace/projects/MIRAW) (accessed on 25 March 2025), and DeepMirTar. All tools were accessed between January and March 2025. Default prediction parameters were used for all three tools, with a prediction probability threshold of ≥0.8 for miTAR.

The final list of candidate miRNAs and their target proteins was used for pathway analysis using IPA [19]. This analysis helped to construct molecular networks and predict the functional impact of the differentially expressed molecules on melanogenesis pathways.

### 2.7. Melanin Content Assay

B16F10 cells were seeded in 24-well plates at a density of 5 × 10^4^ cells/well and allowed to adhere overnight. The following day, the culture medium was replaced with treatment medium containing UCMSC-derived EVs, 10% CBP, or a combination with 0.1 μM α-melanocyte-stimulating hormone (α-MSH) for the melanin-inhibition assay. In parallel, cells treated with 0.1 μM α-MSH alone served as the positive control for the melanin-promoting assay. All treatments were performed in biological triplicates, and the entire assay was repeated on three independent experimental days.

After 72 h of treatment, cells were harvested and lysed with 1 N NaOH at 80 °C for 1 h to solubilize melanin. The optical density (OD) of each lysate was measured at 405 nm using a microplate reader. Melanin content was quantified by comparing sample OD_405_ values with those of a melanin standard curve. The intracellular melanin level was normalized to the total cell number and expressed as melanin content per cell.

### 2.8. RT-qPCR Analysis

Total RNA was extracted from B16F10 cells using the Quick-RNA Miniprep Kit (Zymo Research, Irvine, CA, USA) following the manufacturer’s instructions. Reverse transcription quantitative PCR (RT-qPCR) was performed using the Power SYBR™ Green RNA-to-CT™ 1-Step Kit (Invitrogen, 4389986) on a LightCycler 480 II system (Roche, Basel, Switzerland). Gene expression was normalized to the housekeeping gene β-actin, and relative expression was calculated using the 2^−ΔΔCt^ method. Primer sequences are provided in Supplementary Table S1.

### 2.9. In Vitro Validation of miRNA Function

To validate the function of candidate miRNAs (50 nM) identified through bioinformatic analysis, B16F10 cells were transfected with miRNA mimics or scrambled RNA controls for 72 h using Lipofectamine™ 3000 (Thermo Fisher Scientific, Waltham, MA, USA) according to the manufacturer’s instructions. After transfection, melanin content was measured as described above to confirm the pro- or anti-melanogenic effect of each miRNA. All transfections were performed in biological triplicate, and the entire experiment was repeated on three independent days.

### 2.10. Statistical and Omics Data Analysis

For the miRNA array and proteomic analyses, all computations were conducted in R (version 4.4.1). Raw miRNA array data were imported and quality-controlled using the oligo and limma packages. Background correction, quantile normalization, and log_2_ transformation were applied prior to statistical testing. The limma framework with empirical Bayes moderation was applied, which is robust to moderate imbalance in sample sizes between comparison groups. Differentially expressed miRNAs between UCMSC-derived EVs and CBP were identified using the limma moderated *t*-test with Benjamini–Hochberg correction for multiple testing. miRNAs with adjusted *p* < 0.05 and |log_2_ fold change| > 1 were considered significantly differentially expressed.

Proteomic data were processed and quality-controlled using the Differential Enrichment analysis of Proteomics data (DEP) package in R. Protein intensities were normalized using variance-stabilizing transformation, and missing values were imputed using random draws from a left-shifted Gaussian distribution as implemented in DEP. Differential protein expression between UCMSC-derived EVs and CBP was determined using empirical Bayes statistics from the limma framework. Proteins with adjusted *p* < 0.05 and |log_2_ fold change| > 0.58 (corresponding to a 1.5-fold change) were considered significantly differentially expressed. A more relaxed fold-change threshold was applied for proteomic data relative to miRNA analysis (|log_2_FC| > 1) to account for the typically narrower dynamic range of protein-level changes.

All visualizations, including volcano plots, PCA, and heatmaps, were generated in R using the ggplot2 package.

For assays, all experiments were independently performed at least three times unless otherwise specified. Data are presented as the mean ± standard deviation (SD). Statistical comparisons between two groups were conducted using an unpaired Student’s *t*-test, and multiple-group comparisons were analyzed by two-way ANOVA followed by Tukey’s post hoc test. Statistical analyses were performed using GraphPad Prism 10 (GraphPad Software, San Diego, CA, USA), and a *p*-value < 0.05 was considered statistically significant.

## 3. Results

### 3.1. Characterization of UCMSCs

UCMSCs exhibited a typical spindle-shaped, fibroblast-like morphology under phase-contrast microscopy (Figure S1A). Flow cytometry analysis demonstrated that UCMSCs expressed canonical MSC surface markers, including CD13, CD29, CD44, CD73, CD90, and CD105, while lacking the expression of hematopoietic and endothelial markers CD14, CD19, CD31, CD34, CD45, and HLA-DR (Figure S1B).

In addition, UCMSCs retained multipotent differentiation capacity, as confirmed by successful osteogenic, adipogenic, and chondrogenic differentiation under lineage-specific induction conditions (Figure S1C–E).

### 3.2. Characterization of EVs from UCMSC and CBP

**Figure 1:**
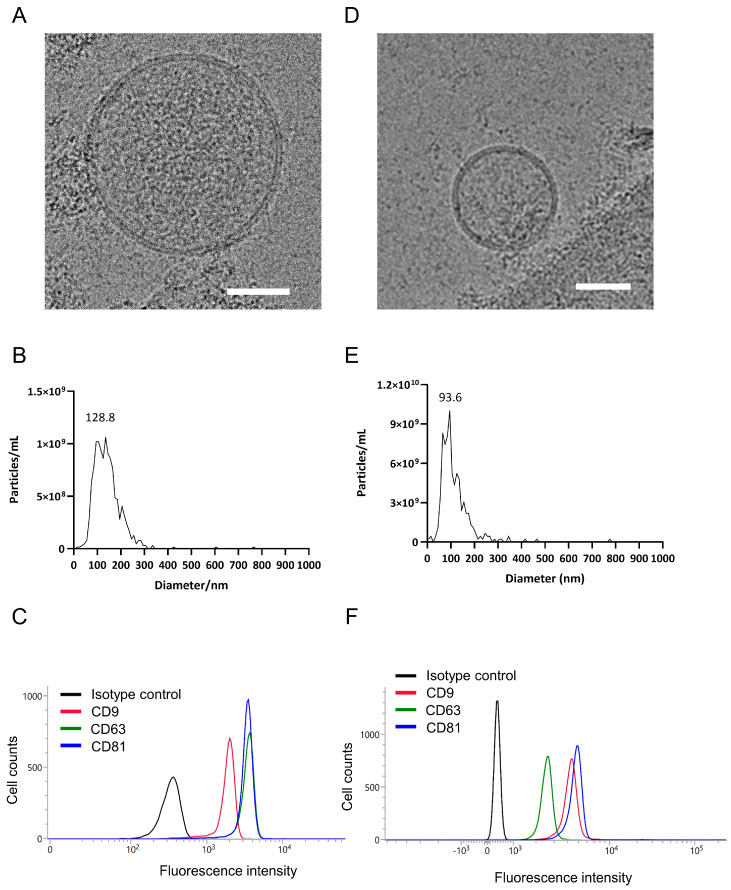
**Characterization of UCMSC-derived EVs and CBP:** (**A**) Cryo-EM images showing the spherical morphology of UCMSC-derived EVs (white scale bar = 50 nm). (**B**) NTA demonstrating the size distribution of UCMSC-derived EVs (a primary size peak at ~128.8 nm). (**C**) Flow cytometry analysis of UCMSC-derived EVs confirmed the expression of exosomal surface markers CD9, CD63, and CD81. (**D**) Cryo-EM images showing the spherical morphology of CBP (white scale bar = 50 nm). (**E**) NTA demonstrating the size distribution of CBP (a primary size peak at ~93.6 nm). (**F**) Flow cytometry analysis of CBP confirmed the expression of EV surface markers CD9, CD63, and CD81.

We next characterized the EVs isolated from human UCMSC-derived EVs and particles present in CBP. Cryo-EM images revealed that both EV populations were spherical in shape, reflecting their native morphology (Figure 1A,D). NTA showed that UCMSC-derived EVs had a primary size peak at ~128.8 nm, whereas CBP peaked at ~93.6 nm; particles present in CBP were slightly smaller, but their particle concentration was approximately 10-fold higher than UCMSC-derived EVs (Figure 1B,E). NTA measurements were performed in biological triplicate and repeated on three independent experimental days. Flow cytometry analysis confirmed that UCMSC-derived EVs were positive for the canonical EV surface markers CD9, CD63, and CD81, while CBP samples also showed detectable signals for these markers (Figure 1C,F). These results indicate the presence of EV-like vesicles in both UCMSC-derived and plasma-derived preparations.

### 3.3. UCMSC-Derived EVs Attenuate Melanogenesis While CBP Enhances It

**Figure 2:**
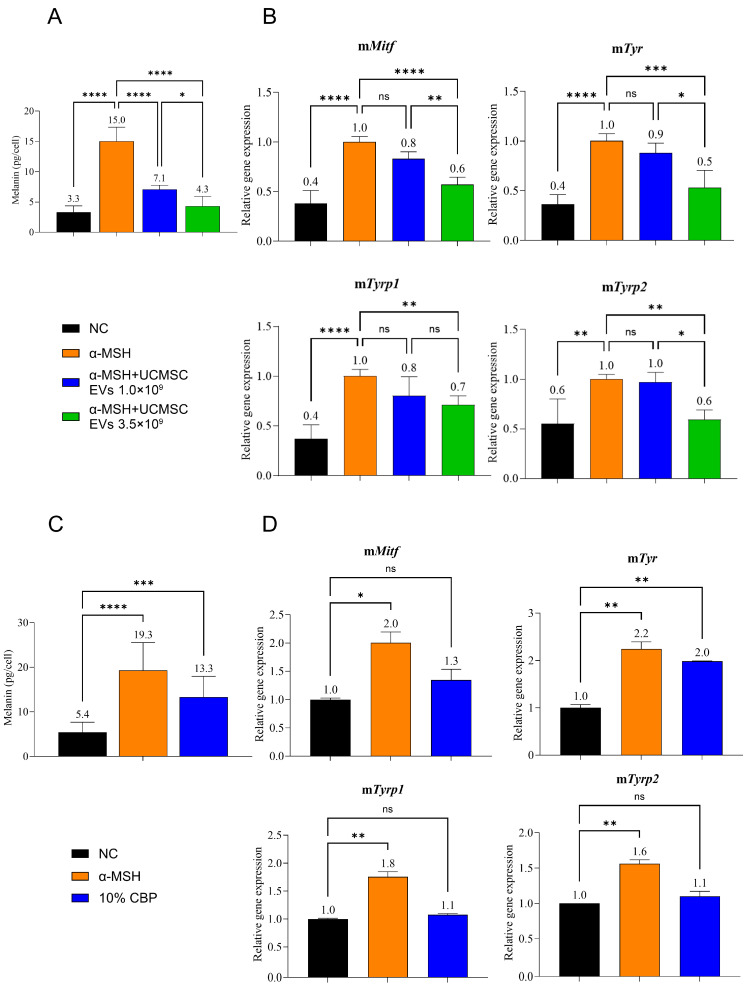
**UCMSC-derived EVs attenuate α-MSH-induced melanogenesis, whereas CBP promotes melanogenesis in B16F10 cells:** (**A**) Melanin content assay for UCMSC-derived EVs. B16F10 cells were cultured for 72 h under four conditions: untreated control, α-MSH alone, α-MSH plus low-dose UCMSC-derived EVs, or α-MSH plus high-dose UCMSC-derived EVs. (**B**) Quantitative PCR of melanogenesis-related genes for UCMSC-derived EVs. After the same treatments, mRNA levels of *Mitf*, *Tyr*, *Tyrp1*, and *Tyrp2* were analyzed by qPCR. (**C**) Melanin content assay for CBP. B16F10 cells were cultured for 72 h under three conditions: untreated control, α-MSH (positive control), and CBP. (**D**) Quantitative PCR of melanogenesis-related genes for CBP. Following the same treatments, mRNA levels of *Mitf*, *Tyr*, *Tyrp1*, and *Tyrp2* were measured by qPCR. * *p* < 0.05, ** *p* < 0.01, *** *p* < 0.001, **** *p* < 0.0001, ns = not significant.

To investigate the functional effects of these EVs on pigmentation, we used the B16F10 melanoma cell line, a standard model for studying melanogenesis. As shown in Figure 2A, treatment with α-MSH significantly increased melanin content compared to the untreated control. Co-treatment with UCMSC-derived EVs led to a dose-dependent reduction in α-MSH-induced melanin production. Consistent with this, RT-qPCR analysis revealed that significant suppression of melanogenesis-related genes (*Mitf*, *Tyr*, *Tyrp1*, and *Tyrp2*) was observed only at the higher EV concentration (3.5 × 10^9^ particles/mL), whereas the lower concentration did not significantly affect gene expression (Figure 2B). These results indicate that the inhibitory effect of UCMSC-derived EVs on melanogenic gene expression is dose-dependent and requires a sufficiently high concentration.

In stark contrast, treatment with CBP resulted in a significant increase in melanin content, even without α-MSH stimulation, an effect comparable to the positive control (α-MSH) (Figure 2C). Consistent with this observation, qPCR results demonstrated that CBP significantly upregulated the basal expression of *Tyr*. Slightly upregulated was also the mRNA level of *Mitf*; however, it was not statistically significant (Figure 2D). These findings clearly demonstrate that UCMSC-derived EVs and CBP exert opposing effects on melanogenesis.

### 3.4. AI-Driven Multi-Omics Integration Pipeline for Candidate miRNA Identification

**Figure 3:**
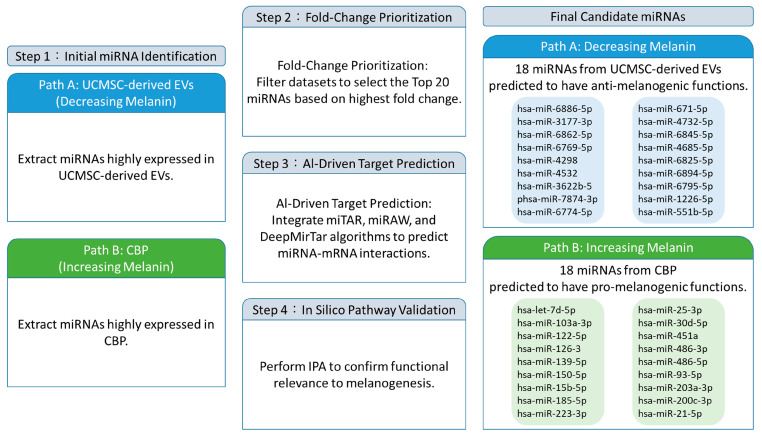
**Bioinformatic pipeline for selecting candidate miRNAs.** The workflow illustrates the process of selecting miRNAs associated with decreasing melanin (anti-melanogenic function) from UCMSC-derived EVs and increasing melanin (pro-melanogenic function) from CBP.

To systematically identify candidate miRNAs potentially involved in melanogenesis, we established an AI-driven bioinformatic workflow integrating differential expression analysis, target prediction, and pathway annotation (Figure 3). This pipeline was designed to prioritize high-confidence regulatory miRNAs by leveraging complementary computational approaches.

First, differentially expressed miRNAs between UCMSC-derived EVs and CBP were identified based on microarray analysis. These miRNAs were then subjected to target prediction using multiple deep learning-based algorithms, including miTAR, miRAW, and DeepMirTar, each of which captures distinct features of miRNA–mRNA interactions. miTAR utilizes a hybrid convolutional and recurrent neural network framework to model sequence patterns, whereas miRAW evaluates full-length miRNA transcripts to detect both canonical and non-canonical binding sites, and DeepMirTar incorporates a broad feature set using stacked autoencoder architectures (Table 1).

To improve prediction robustness, candidate miRNAs were prioritized when supported by at least two of the three prediction tools. The predicted target genes were subsequently cross-referenced with curated melanogenesis-related gene sets from established databases, including KEGG and Gene Ontology.

Finally, the selected miRNA–target pairs were subjected to IPA to evaluate their potential functional relevance within known biological pathways. This integrative approach enabled the prioritization of candidate miRNAs for downstream validation while minimizing bias associated with single-method predictions.

### 3.5. Differential miRNA and Protein Profiles in UCMSC-Derived EVs and CBP

**Figure 4:**
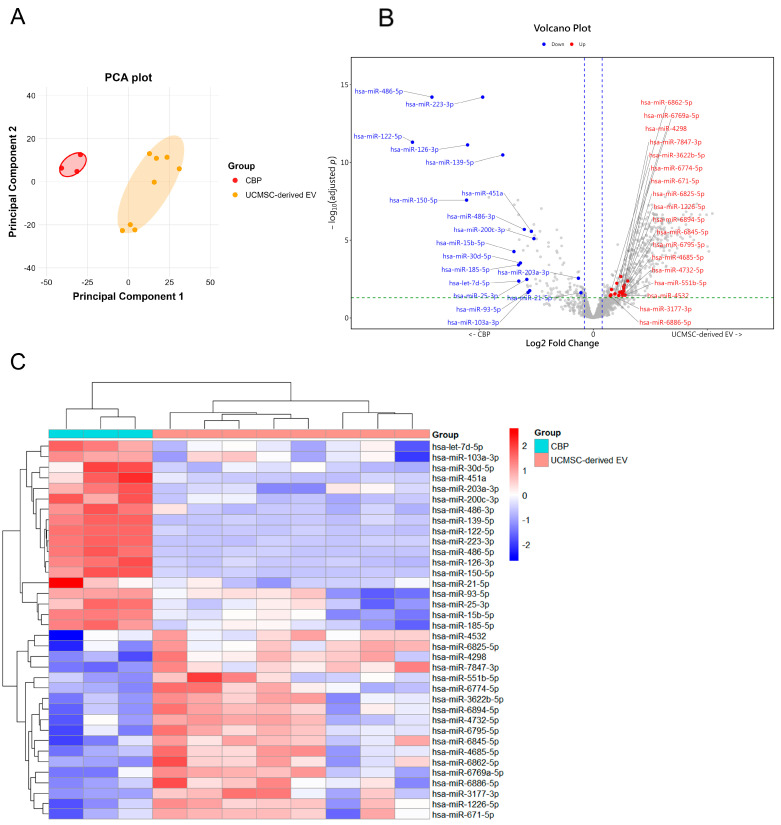
**Differential miRNA profiles of UCMSC-derived EVs and CBP:** (**A**) PCA plot showing distinct clustering of miRNA expression profiles from UCMSC-derived EVs and CBP. (**B**) Volcano plot showing differentially expressed miRNAs between UCMSC-derived EVs and CBP. Each dot represents one miRNA. Gray dots indicate all detected miRNAs, whereas the labeled red and blue dots represent the 36 predicted functional miRNAs with increased and decreased expression, respectively. The x-axis shows the log2 fold change, and the y-axis shows the −log10 of the adjusted *p*-value. The blue dashed vertical lines indicate the fold-change cutoff (|log2FC| ≥ log2(1.5)), and the green dashed horizontal line indicates the statistical significance threshold (adjusted *p* < 0.05). (**C**) Heatmap showing differentially expressed miRNAs between UCMSC-derived EVs and CBP.

PCA of the miRNA expression profiles showed a clear separation between the UCMSC-derived EVs and CBP groups, indicating distinct miRNA signatures (Figure 4A). Volcano plots and heatmaps further highlighted the differentially expressed miRNAs, revealing a set of miRNAs significantly different between UCMSC-derived EVs compared to CBP (Figure 4B,C). A similar distinct clustering was observed in the proteomic data, with PCA separating the two groups and volcano plots identifying differentially expressed proteins (Figure S2A–C).

To obtain a global overview of the biological processes associated with the differential molecular profiles, we performed unbiased pathway enrichment analyses based on both miRNA-predicted targets and proteomic data. These analyses revealed enrichment of multiple biological themes, including oxidative stress response, inflammatory signaling, extracellular matrix remodeling, angiogenesis, and pigmentation-related pathways. Among these, melanogenesis emerged as the most consistently enriched process across both omics layers. Notably, both miRNA target predictions and differentially expressed proteins converged on key regulators within the MITF–TYR signaling axis, which is central to melanin synthesis. In addition, the directionality of these molecular changes was coherent between datasets, with UCMSC-derived EV-associated molecules predominantly linked to the suppression of melanogenic pathways, whereas CBP-associated molecules were associated with the activation of these pathways. Based on this cross-omics consistency and biological relevance, we prioritized melanogenesis as the primary focus for subsequent functional validation and mechanistic analysis.

### 3.6. Identification and Validation of Melanogenesis-Modulating miRNAs

**Figure 5:**
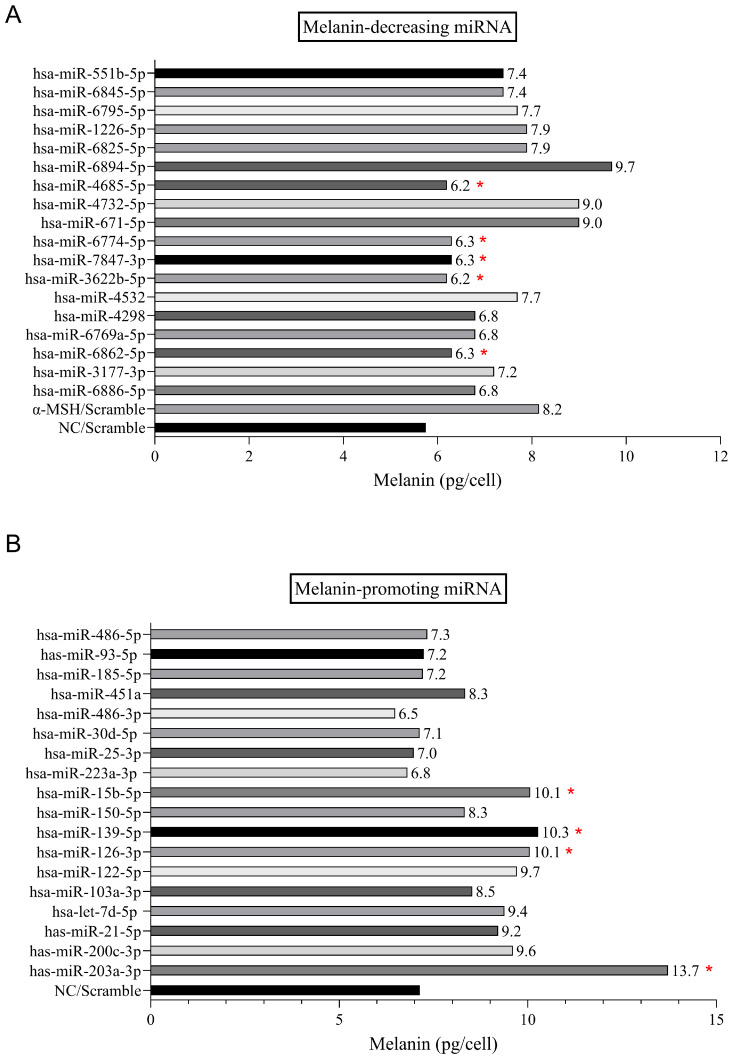
**Identification and in vitro validation of miRNAs that modulate melanin production:** (**A**) List of 18 candidate miRNAs identified from UCMSC-derived EVs predicted by IPA to suppress melanogenesis. (**B**) List of 18 candidate miRNAs identified from CBP predicted by IPA to enhance melanogenesis. Subsequent in vitro validation by transfecting B16F10 cells with representative miRNA mimics confirmed their respective inhibitory or stimulatory effects on melanin content. Asterisks indicate significant changes in melanin levels for selected miRNAs.

From differential miRNA analysis (adjusted *p* < 0.05, |log_2_FC| > 1), we defined two 18-miRNA panels: an anti-melanogenic set enriched in UCMSC-derived EVs and reduced in CBP, and a pro-melanogenic set enriched in CBP and reduced in UCMSC-derived EVs. Cross-tool predictions (miTAR, miRAW, DeepMirTar) and secondary database analyses converged on core melanogenic regulators, including *Mitf*, *Tyr*, *Tyrp1*, and *Tyrp2*, as well as their upstream signaling nodes. Representative miRNAs from each group were synthesized as mimics and transfected into B16F10 cells for functional validation. As hypothesized, mimics enriched in UCMSC-derived EVs significantly decreased melanin content, whereas those enriched in CBP increased it, thereby confirming the predicted inhibitory or stimulatory effects on melanogenesis (Figure 5A,B). Bars marked with asterisks indicate significant changes in melanin levels, highlighting miRNAs with potential melanogenic activity.

### 3.7. Integrated Network Analysis of miRNAs and Proteins

**Figure 6:**
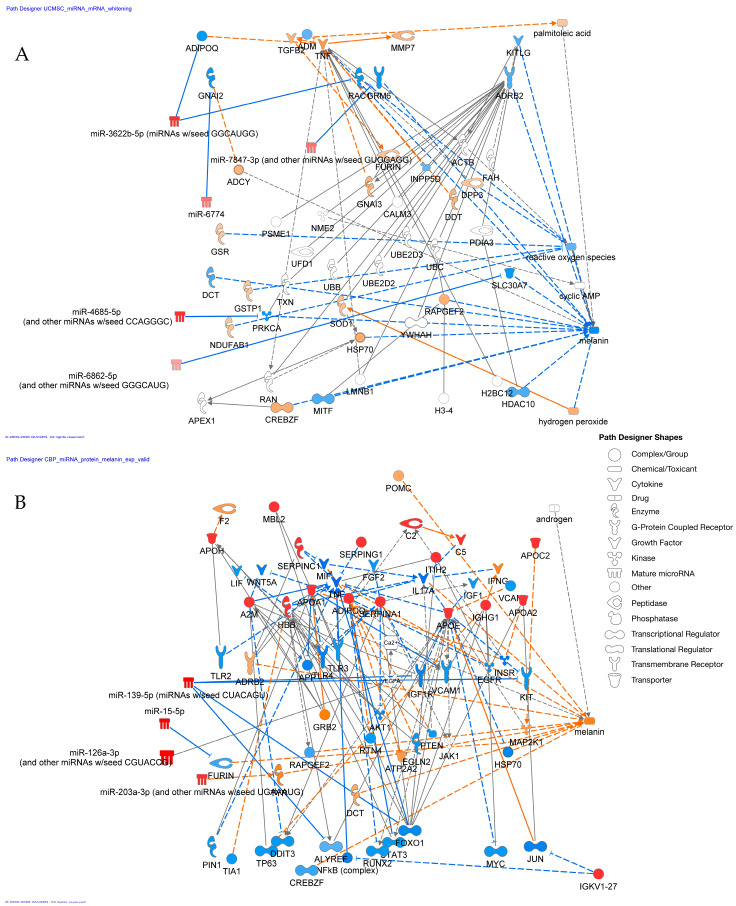
**Integrated network analysis of melanogenesis-associated miRNAs and proteins:** (**A**) Network showing highly expressed miRNAs and proteins in UCMSC-derived EVs predicted to decrease melanin production. (**B**) Network showing highly expressed miRNAs and proteins in CBP predicted to increase melanin production. The networks were generated using IPA (QIAGEN Inc.).

Finally, we integrated the miRNA and proteomics data using IPA to construct regulatory networks associated with melanogenesis. The analysis revealed a distinct network for each EV type. For UCMSC-derived EVs, the network highlighted highly expressed miRNAs and proteins that converge on pathways known to decrease melanin production (Figure 6A). Conversely, the network for CBP showed that its unique cargo of miRNAs and proteins is associated with pathways that increase melanin synthesis (Figure 6B). These networks provide a molecular blueprint explaining the opposing functional effects of the two EV populations.

## 4. Discussion

The therapeutic and cosmetic potential of EVs has drawn considerable interest, particularly those derived from perinatal sources like the umbilical cord. While often grouped together, our study highlights a critical finding: EVs from different compartments of the umbilical cord unit—specifically, the cord tissue-derived UCMSCs and CBP—possess not just different, but functionally opposite, effects on a key dermatological process, melanogenesis. We demonstrated that UCMSC-derived EVs act as inhibitors of melanin synthesis, suggesting a potential for skin-lightening applications, whereas CBP acts as a promoter, indicating a possible role in treating hypopigmentation or in applications like preventing hair graying.

Our initial characterization confirmed the quality and identity of the isolated EVs, consistent with the standards in the field [1]. In addition, vesicle-like particles were detectable in CBP preparations, which is expected since plasma naturally contains EVs together with soluble circulating components. The functional dichotomy was clearly established in B16F10 cells, where UCMSC-derived EVs dose-dependently suppressed melanin production and the mRNA expression of master regulator *Mitf* and its downstream targets (*Tyr*, *Tyrp1*, and *Tyrp2*). This finding aligns with several reports suggesting that MSC-derived exosomes can inhibit melanogenesis, potentially through the transfer of anti-melanogenic miRNAs or proteins [7,8]. The mechanism often involves targeting the MITF signaling axis, which our qPCR data strongly support.

Conversely, the stimulatory effect of CBP on melanogenesis is a more novel finding. Cord blood is a rich source of growth factors and signaling molecules, and it appears this pro-growth environment is reflected in the molecular cargo present in CBP. The upregulation of MITF and melanogenic enzymes following CBP treatment suggests that they activate the core pigmentation machinery. This pro-melanogenic activity could be beneficial in conditions characterized by a loss of melanocytes or their function, such as vitiligo, or in cosmetic applications aimed at restoring natural hair color.

The core of our study was to move beyond functional observation to mechanistic insight. By employing an integrative omics approach, we successfully linked the functional differences to distinct molecular signatures. The clear separation of miRNA and protein profiles between UCMSC-derived EVs and CBP (Figure 4 and Figure S2) provided the foundation for this analysis. The use of a multi-pronged, AI-driven bioinformatic strategy (Table 1) was instrumental in navigating the complexity of miRNA target prediction. Relying on a single algorithm can be limiting, as different models have different strengths; for example, miTAR excels with raw sequence data, while miRAW is adept at finding non-canonical sites [15,16]. By combining these predictive tools with the knowledge-based curation of IPA, we were able to generate a high-confidence list of candidate miRNAs, which were subsequently validated in vitro (Figure 5).

The integrated network analysis (Figure 6) provides a powerful visualization of the molecular machinery at play. In UCMSC-derived EVs, the network likely includes miRNAs that directly target key melanogenic mRNAs (e.g., *Mitf*, *Tyr*) and proteins that may interfere with melanosome maturation or transfer. For example, studies have shown that specific miRNAs like miR-181a-5p and miR-199a from amniotic stem cells can suppress MITF [14]. Our data suggest a similar mechanism for UCMSC-derived EVs. In contrast, the CBP-associated network points to a different set of molecules that activate upstream signaling pathways (e.g., Wnt/β-catenin or cAMP pathways) that converge on MITF activation or proteins that directly support melanosome function.

Our findings align with and extend a growing body of evidence implicating miRNAs as central regulators of melanogenesis through diverse mechanisms. MITF, the master transcription factor governing melanogenic gene expression, is a direct target of multiple miRNAs. miR-25 and miR-508-3p were among the first to be shown to suppress MITF expression in melanocytes, with miR-25 simultaneously reducing MITF protein, TYR, and TYRP1 levels [13]. Subsequently, miR-137 and miR-148a were identified as negative regulators of MITF in melanoma cells, and miR-141-3p and miR-200a-3p were demonstrated to directly target the 3′-UTR of *Mitf* in B16F10 melanocytes, with their overexpression suppressing both melanogenesis and TYR activity; notably, topical application of these miRNAs inhibited melanin biosynthesis in 3D reconstructed human skin [20]. In addition to direct *MITF* targeting, miR-27a-3p has been shown to inhibit melanogenesis in human epidermal melanocytes by suppressing Wnt3a, a ligand of the Wnt/β-catenin signaling pathway that converges on *MITF* transcriptional activation [21]. Beyond MITF, other melanogenic enzymes are also subject to miRNA regulation: miR-434-5p and miR-330-5p directly target TYR to reduce melanin synthesis, with miR-330-5p achieving depigmentation without affecting cell proliferation [13].

The role of exosome-mediated miRNA transfer in intercellular pigmentation regulation has been increasingly recognized. Lo Cicero et al. demonstrated that exosomes released by keratinocytes directly modulate melanocyte pigmentation in a phototype-dependent manner, identifying exosomal miR-203 and miR-3196 as pro-melanogenic mediators that enhance *TYR* expression and melanin content [22]. Keratinocyte-derived exosomal miR-330-5p was shown to suppress melanogenesis by targeting *TYR* in recipient melanocytes, establishing a paracrine anti-melanogenic axis [23]. In vitiligo lesional skin, downregulation of exosomal miR-200c from keratinocytes was found to suppress melanogenesis via de-repression of *SOX1*, providing mechanistic insight into pigmentation loss [24]. Kim et al. reported that reduced exosomal miR-675 from keratinocytes contributes to melanogenesis regulation through direct targeting of MITF [25]. More recently, Yoon et al. performed comprehensive profiling of UVB-irradiated keratinocyte-derived exosomal miRNAs and identified miR-644a, miR-365b-5p, and miR-29c-3p as pro-melanogenic mediators, while miR-18a-5p, miR-197-5p, and miR-4281 exhibited anti-melanogenic activity [26]. Additionally, Jeon et al. profiled miRNAs in B16F10 melanoma cell-derived exosomes and identified miR-21a-5p as a potential facilitator of melanin synthesis [27].

In the context of MSC-derived EVs, Wang et al. reported that human amniotic stem cell-derived exosomal miR-181a-5p and miR-199a inhibit melanogenesis by targeting *MITF* and promote melanosome degradation through autophagy activation, respectively [14]. These findings are consistent with our observation that UCMSC-derived EVs carry anti-melanogenic miRNA cargo and provide a mechanistic precedent for the functional effects we observed. Conversely, the pro-melanogenic activity of CBP is consistent with the presence of soluble factors and vesicular cargo enriched in growth factors and signaling molecules known to activate the MITF/TYR axis. Collectively, these studies establish that the miRNA content of extracellular vesicles from diverse cellular sources plays a functionally significant role in melanogenesis regulation, supporting our integrative approach of profiling EV-associated and plasma-associated miRNAs to identify source-specific melanogenic modulators.

This study has several implications. First, it underscores the importance of source selection in the development of EV-based therapeutics. It is not sufficient to refer to “cord-derived EVs”; the specific origin (tissue vs. blood) is critical, as it dictates biological function. Second, our validated lists of pro- and anti-melanogenic miRNAs provide a rich resource for future research and development. These miRNAs could be developed as standalone therapeutics (e.g., as mimics or inhibitors) or used to engineer “designer” EVs with enhanced potency for a desired effect [28]. For example, enriching EVs with the anti-melanogenic miRNAs from UCMSCs could lead to a more effective skin-lightening agent.

Limitations of this study include its reliance on an in vitro mouse melanoma cell line. Future work should validate these findings in human primary melanocytes and keratinocyte co-culture systems, as well as in 3D skin models and eventually in vivo. We note that the sample size between groups was not balanced, which may introduce variability; however, the statistical methods applied in this study are designed to mitigate such effects. Furthermore, while we validated the function of representative miRNAs, a full elucidation of the complex interplay between all the differentially expressed miRNAs and proteins is required to build a complete mechanistic picture. In addition, because CBP was used as a plasma preparation rather than a purified EV fraction, the observed biological effects likely reflect the combined contribution of EVs and soluble plasma factors.

## 5. Conclusions

In conclusion, our study provides compelling evidence that UCMSC-derived EVs and CBP exert opposing effects on melanogenesis, with UCMSC-derived EVs being inhibitory and CBP showing stimulatory activity. This functional divergence is driven by distinct miRNA and protein cargos. Through advanced, AI-driven omics analysis, we identified and validated specific miRNA candidates responsible for these effects and mapped their interactions within the broader melanogenesis regulatory network. These findings not only deepen our understanding of EV-mediated pigmentation control but also pave the way for the rational design of targeted, source-specific EV therapies for a range of dermatological and cosmetic applications.

## Figures and Tables

**Table 1:** AI-driven screening of miRNA–target gene pairs for melanogenesis regulation.

Method	Prediction Principle	Example Candidate miRNAs	Predicted Target Genes (Examples)	Data Source/Reference	Selection Rationale & Process
miTAR	Hybrid deep learning (CNN + RNN) model analyzing raw sequence data for spatial and sequential features.	hsa-miR-6862-5p, hsa-miR-3622b-5p	*MITF*, *TYR*	Gu et al., 2021 [15]	Selected for its high accuracy in predicting miRNA–target interactions without relying on pre-defined features. Used to generate an initial broad list of potential interactions. Target predictions were cross-validated with miRAW and DeepMirTar; miRNA–target pairs predicted by ≥2 tools were retained as high-confidence candidates.
miRAW	Deep learning model analyzing the entire miRNA transcript, enabling detection of non-canonical binding sites.	hsa-miR-203a-3p, hsa-miR-139-5p	*TYRP1*, *DCT (TYRP2)*	Pla et al., 2018 [16]	Chosen to complement seed-based methods and identify novel regulatory interactions. Targets were filtered for known roles in pigmentation pathways. Of the 20 candidates per panel, target overlap with miTAR and DeepMirTar was used to prioritize high-confidence interactions.
DeepMirTar	Deep learning (stacked de-noising auto-encoders) using a wide range of expert-designed and raw data features.	hsa-miR-7847-3p, hsa-miR-4685-5p	*SOX10*, *PAX3*	Wen et al., 2018 [18]	Utilized for its comprehensive feature-based approach to refine the candidate list and improve prediction confidence for both canonical and non-canonical sites. Final high-confidence set comprised 18 miRNAs per panel with ≥2-tool agreement on melanogenesis-relevant targets.
IPA	Knowledge-based analysis of curated literature to map molecules to pathways, functions, and diseases.	Top-ranked miRNAs selected for validation	Network of genes in the melanogenesis signaling pathway	QIAGEN [19]	Final step to functionally annotate the high-confidence miRNA candidates from the AI tools. miRNAs were prioritized based on their predicted upstream or downstream effects on the “Melanogenesis Signaling” canonical pathway.

## Data Availability

All data supporting the findings of this study are included in the article and its Supplementary Files. Additional information is available from the corresponding author upon reasonable request.

## Supplementary Materials

The following supporting information can be downloaded at: https://www.mdpi.com/article/10.3390/cimb48040391/s1.
