# Supplementary material for: Omic Profiling of Extracellular Vesicles from Two Cord-Related Sources Reveals Divergent Effects on Melanogenesis
Source: Curr Issues Mol Biol. 2026 Apr 10;48(4):391. doi: 10.3390/cimb48040391 (PMC13115239; doi:10.3390/cimb48040391)

Supplementary Table S1. Primer sequences for qRT-PCR.

No	Primer name	Primer Sequence
1	mouse β-actin_F	CATTGCTGACAGGATGCAGAAGG
2	mouse β-actin_R	TGCTGGAAGGTGGACAGTGAGG
3	mouse TYRP1_F	AGCCACAGGATGTCACTCAGTG
4	mouse TYRP1_R	GCAGGGTCATATTTTCCCGTGG
5	mouse TYRP2_F	GCAAGATTGCCTGTCTCTCCAG
6	mouse TYRP2_R	CTTGAGAGTCCAGTGTTCCGTC
7	mouse TYR_F	CAGGCTCCCATCTTCAGCAGAT
8	mouse TYR_R	ATCCCTGTGAGTGGACTGGCAA
9	mouse MITF_F	GATCGACCTCTACAGCAACCAG
10	mouse MITF_R	GCTCTTGCTTCAGACTCTGTGG

Supplementary Figure S1. Characterization of UCMSCs.

A

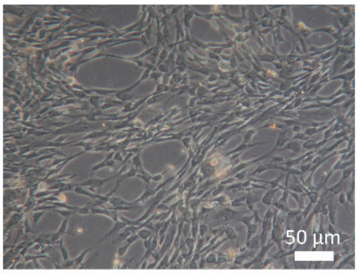

B

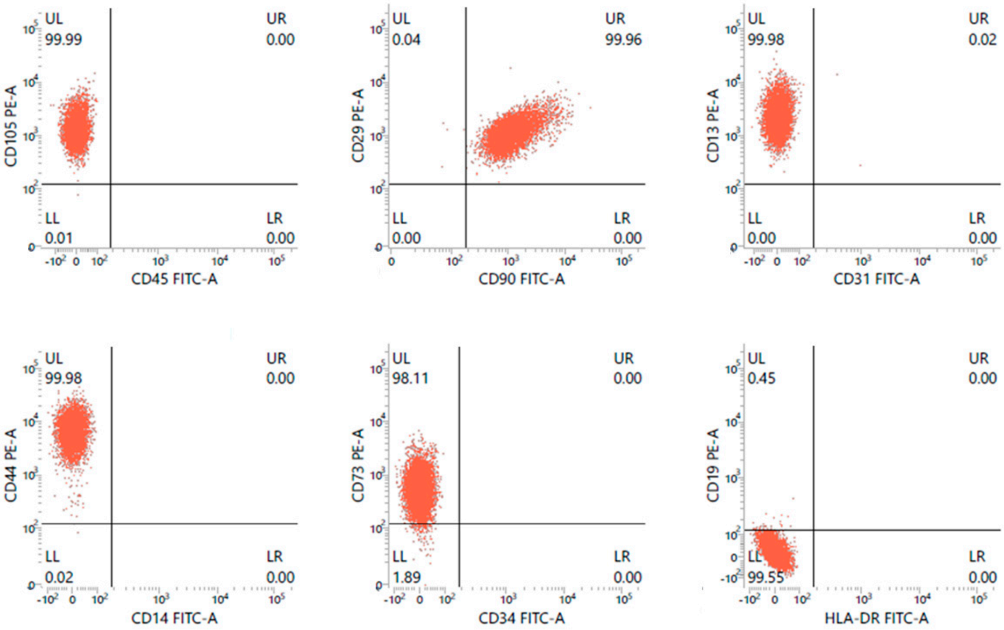

C

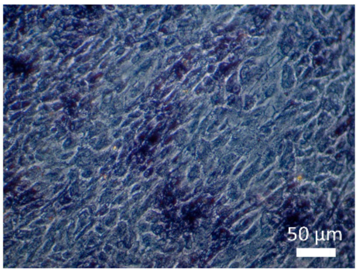

D

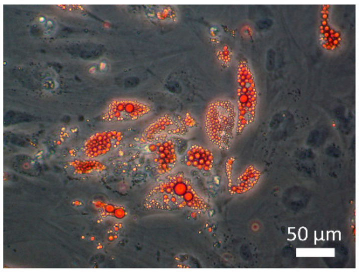

E

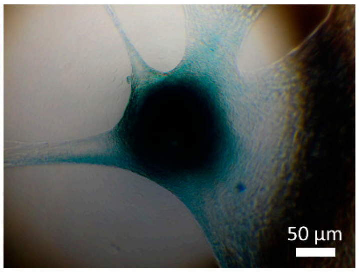

Supplementary Figure S2. Differential protein profiles of UCMSC-derived EVs and CBP.

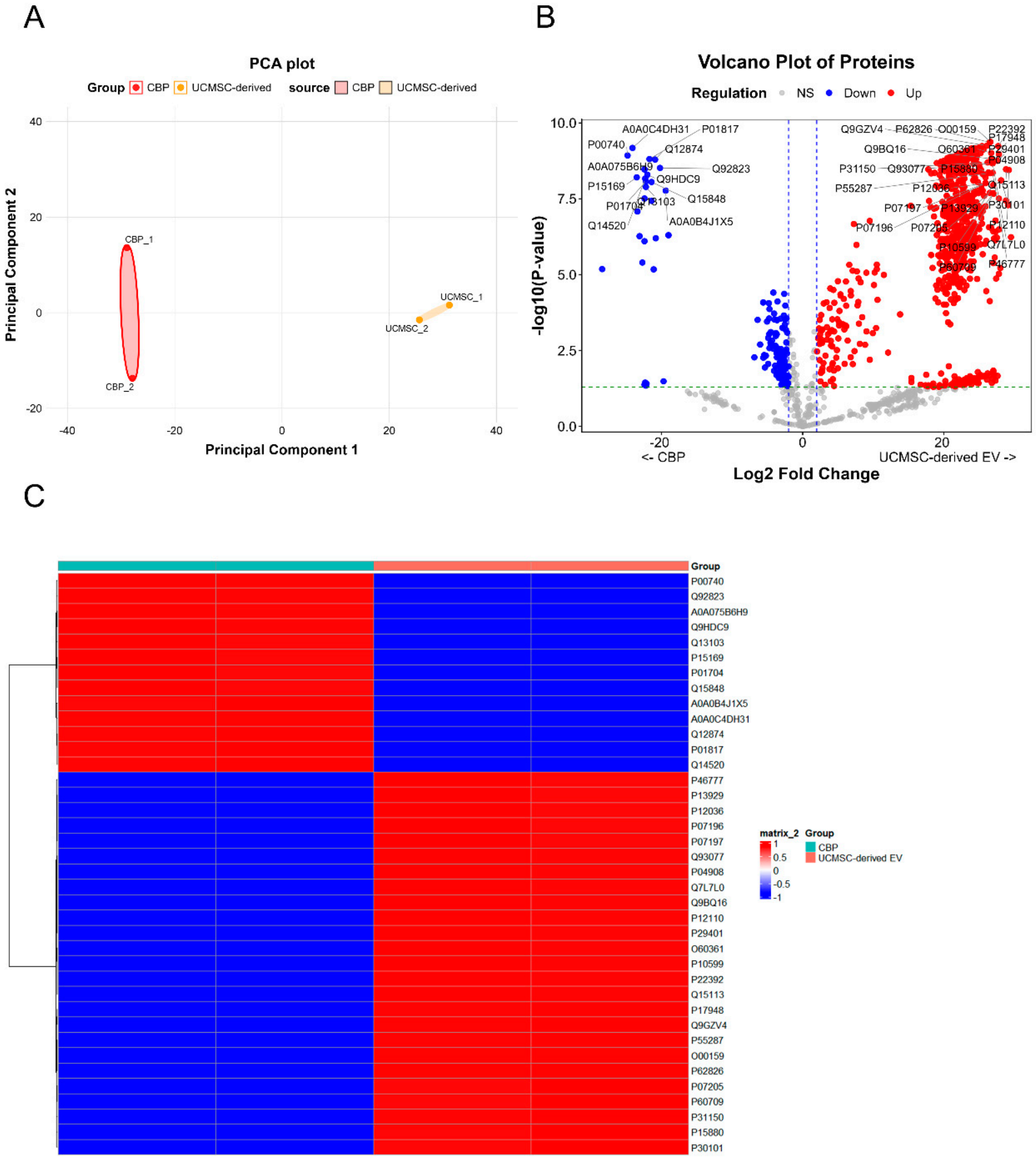

Supplement: Supplementary file 1 [file cimb-48-00391-s001.zip › cimb-4193958-supplementary.pdf]
